# *Porphyromonas Gingivalis* and *E-coli* Induce Different Cytokine Production Patterns in Pregnant Women

**DOI:** 10.1371/journal.pone.0086355

**Published:** 2014-01-22

**Authors:** Marijke M. Faas, Alina Kunnen, Daphne C. Dekker, Hermie J. M. Harmsen, Jan G. Aarnoudse, Frank Abbas, Paul De Vos, Maria G. Van Pampus

**Affiliations:** 1 Division of Medical Biology, Department of Pathology and Medical Biology, University Medical Center Groningen and University of Groningen, Groningen, The Netherlands; 2 Department of Periodontology, Center for Dentistry and Oral Hygiene, University Medical Center Groningen and University of Groningen, Groningen, The Netherlands; 3 School of Health Care Studies, Hanze University of Applied Sciences Groningen, Groningen, The Netherlands; 4 Department of Medical Microbiology, University Medical Center Groningen and University of Groningen, Groningen, The Netherlands; 5 Department of Obstetrics and Gynecology, University Medical Center Groningen and University of Groningen, Groningen, The Netherlands; University of Missouri-Kansas City, United States of America

## Abstract

**Objective:**

Pregnant individuals of many species, including humans, are more sensitive to various bacteria or their products as compared with non-pregnant individuals. Pregnant individuals also respond differently to different bacteria or their products. Therefore, in the present study, we evaluated whether the increased sensitivity of pregnant women to bacterial products and their heterogeneous response to different bacteria was associated with differences in whole blood cytokine production upon stimulation with bacteria or their products.

**Methods:**

Blood samples were taken from healthy pregnant and age-matched non-pregnant women and ex vivo stimulated with bacteria or LPS from *Porphyromonas Gingivalis* (Pg) or *E-coli* for 24 hrs. TNFα, IL-1ß, IL-6, IL-12 and IL-10 were measured using a multiplex Luminex system.

**Results:**

We observed a generally lower cytokine production after stimulation with Pg bacteria or it’s LPS as compared with *E-coli* bacteria. However, there was also an effect of pregnancy upon cytokine production: in pregnant women the production of IL-6 upon Pg stimulation was decreased as compared with non-pregnant women. After stimulation with *E-coli*, the production of IL-12 and TNFα was decreased in pregnant women as compared with non-pregnant women.

**Conclusion:**

Our results showed that cytokine production upon bacterial stimulation of whole blood differed between pregnant and non-pregnant women, showing that the increased sensitivity of pregnant women may be due to differences in cytokine production. Moreover, pregnancy also affected whole blood cytokine production upon Pg or *E-coli* stimulation differently. Thus, the different responses of pregnant women to different bacteria or their products may result from variations in cytokine production.

## Introduction

Periodontal diseases are a group of diseases caused by inflammation and destruction of the supporting and investing structures of the teeth and the periodontal tissues [1]. The infection in the oral cavity can lead to systemic inflammation resulting in adverse medical outcomes. Indeed, associations between periodontal disease and cardiovascular disease (CVD) [2], stroke [3], glycemic control in diabetes [4] and rheumatoid arthritis [5] have been found. It has also become evident that periodontitis during pregnancy may result in adverse outcome; the presence of periodontitis during pregnancy has been associated with intrauterine growth restriction (IUGR) [6] or preterm birth [7]. Although there are many bacterial species present in the infected oral cavity, *Porphyromonas gingivalis* (Pg) has most frequently been associated with systemic disease [8]. This is probably due to the fact that this bacterium has the capacity to disseminate into the peripheral circulation and cause inflammation at other sites [8].

The mechanism responsible for the association between periodontitis and pregnancy complications remains to be unraveled, but a route via the peripheral circulation to the placenta is likely to be involved [9], [10]. Plausibly, also activation of the systemic inflammatory response by oral bacteria, such as Pg or their products, is involved. It is well known that pregnancy is a proinflammatory condition [11], with phenotypically activated monocytes and changes in monocyte function, such as cytokine production [12], [13].Although the exact stimulus for the activated inflammatory cells is unknown, it is thought that factors shed from the placenta activate the inflammatory cells [14]–[16]. Therefore, it seems likely that during pregnancy the systemic inflammatory response to bacteria and their products is different as compared with this response in non-pregnant women. Indeed, pregnant individuals, including humans, are much more sensitive to one of the products of the *E-coli* bacterium, lipopolysaccharide (LPS), than non-pregnant individuals [17]. For instance it has been shown that infusion of a low dose of *E-coli* LPS (1.0 µg/kgbw) induced hypertension and proteinuria in pregnant animals only; non-pregnant rats did not develop these signs [18].

Interestingly, infusion of a low dose of Pg LPS into pregnant rats in identical circumstances induced hypertension, but not proteinuria. Moreover, while only slightly increased doses of *E-coli* LPS induced hypotension, maternal illness and resorption of most of the fetuses [18], increasing doses of Pg LPS did not induce more severe effects than the low dose of Pg LPS Kunnen [19]. This suggests that pregnant individuals are not only more sensitive to bacterial products, but also that the sensitivity of pregnant individuals to different bacteria or their products differs. In the present study, we hypothesized that the increased sensitivity of pregnant women to bacterial products, and the different sensitivities of pregnant women to different bacterial products could be due to differences in cytokine production of leukocytes upon stimulation of whole blood with bacterial products. To this end, we compared cytokine production following stimulation of whole blood of pregnant and non-pregnant women with Pg or *E-coli* bacteria and their LPS and measured production of the proinflammatory cytokines TNFα, IL-1β, IL-12 and IL-6 as well as the anti-inflammatory IL-10.

## Materials and Methods

### Experimental Design

To compare whole blood cytokine production in non-pregnant and pregnant women following stimulation with Pg or *E-coli* bacteria or their LPS, we stimulated whole blood of non-pregnant and pregnant women with bacteria of Pg or *E-coli* or their LPS. After 24 hrs. of stimulation, we measured the production of pro-inflammatory and anti-inflammatory cytokines in the plasma using a multiplex Luminex system.

### Subjects

This study was approved by the Medical Ethical committee (approval no. 2008/168) at the University Medical Center Groningen, and a written informed consent was obtained from each subject before participation.

Participants (pregnant and age-matched non-pregnant, healthy Caucasian women between 20 and 40 years) were recruited from the Department of Obstetrics and Gynecology, University Medical Center Groningen or recruited from the hospital staff. Exclusion criteria for both groups were: smoking, pre-pregnancy BMI<18 or >25, hypertension, chronic diseases, flu-like symptoms or fever, treatment with antibiotics within 14 days prior to blood sampling or an Dutch Periodontal Screening index (DPSI) score of 3+ or 4 after periodontal screening, which is indicative for destructive periodontal disease [20]. Furthermore, pregnant women were checked until the end of pregnancy and no pregnancy complications were observed.

Whole blood (10 ml; lithium-heparin vacutainer tube (Becton Dickinson, Rutherford, NJ)) was obtained by venous puncture from 16 primigravid women at 30 weeks of gestation (range 28–32 weeks) and from 15 nulligravid women with regular menstrual cycles (26–32 days) in their follicular phase (day 8–10), to minimize variations due to hormonal changes.

### Bacteria

*E-coli* ATCC 25922 was grown on 5% sheep blood agar plates (Mediaproducts Groningen, The Netherlands) in air with 5%CO_2_ at 37°C for 1 day. *P. gingivalis* ATCC 33277 (A.J. van Winkelhoff, Department of Oral Microbiology, Academic Center for Dentistry Amsterdam, The Netherlands) was grown on Brucella blood agar (Mediaproducts), supplemented with 5% sheep blood, 5 mg/L hemin and 1 mg/L menadione in an anaerobic chamber with 5%CO_2_, 10%H_2_ and 85%N_2_ at 37°C. After 1 day (*E-coli*) or 4–7 days (Pg), one bacterial colony was inoculated in Todd-Hewitt-broth (BBL Microbiology Systems), supplemented with hemin (5 mg/L), menadione (5 mg/L) and glucose (2 mg/L) for one day (*E-coli*) or one week (Pg). The bacterial cultures were harvested by centrifugation at 2773 g for 10 minutes at 4°C. The pellet was washed twice in phosphate-buffered saline (PBS). The number of bacteria was evaluated by means of a microscope after gram-staining and resuspended in PBS at a concentration corresponding of approximately 1×10^8^ bacteria/ml and stored at −80°C.

### Lipopolysaccharides

*P. gingivalis* LPS ATCC 33277 (Ultra-Pure, Cat.#: tlrl-pglps, Lot.#: 28-06-PGLPS, InvivoGen, San Diego, USA); *E-coli* LPS (055:B5, BioWhittaker, Walkersville MD, USA).

### Stimulation of Whole Blood with Bacteria and LPS

After sampling, 250 µl of blood was mixed with 250 µl of bacterial cultures of *E-coli* or Pg (final numbers: 5×10^7^ bacteria/ml). A further 250 µl of blood was mixed with 250 µl RPMI (Invitrogen, California, USA) and LPS (*E-coli* or Pg) was added (final concentration: 2 µg/ml). The doses of bacteria and LPS were chosen based on a previous study from our lab, since these doses showed large differences in induction of cytokines between *E-coli* bacteria and LPS and Pg bacteria and LPS [21]. Negative controls were incubated in the absence of bacteria or LPS. Samples were incubated for 24 h at 37°C in a 5%CO_2_ humidified atmosphere. After stimulation, all samples were pipetted into 1.5 ml eppendorf tubes and centrifuged for 10 minutes at 316 g (4°C). The plasma was centrifuged again for 5 minutes at 1972 g (4°C) and frozen at −80°C.

### Determination of Plasma Cytokine Production

Cytokine levels in whole blood were measured using a Bio-Plex™ premixed cytokine assay, human 5-plex group I; cat. #: M50019PLCW, control 5016683 (Bio-Rad Laboratories, Hercules, USA), to measure TNFα, IL-1β, IL-6, IL-10 and IL-12(p70), according to the manufacturers instruction manual. Raw data (mean fluorescence intensity, MFI) were analyzed using STarStation V2.3.

### Toll Like Receptor (TLR) Labeling

Immediately after sampling, 500 µl of whole blood was mixed with 500 µl of RPMI and incubated with PerCp–labeled mouse-anti-human-CD14 (clone TüK4; Invitrogen Corporation, Breda, The Netherlands) together with FITC-labeled mouse-anti-human-TLR2 (clone TL2.1; eBioscience, Breda, The Netherlands) and PE-labeled mouse-anti-human-TLR4 (clone HTA 125; eBiosciences), or with anti-CD14 together with TLR2 and TLR4 isotype controls for 30 minutes at room temperature (RT) in the dark. After 5 minutes incubation with lysing buffer (Becton Dickinson, CA, USA) at RT in the dark, tubes were centrifuged (5 minutes at 467 g) and aspirated. After washing with washing buffer (PBS with 0.5% bovine serum albumin and 0.1% sodium azide), cells were fixed with 0.5% paraformaldehyde and kept at 4°C in the dark until flow cytometry, within 24 h after labeling.

### Flow Cytometry

Cells were analyzed by flow cytometry (FACSCalibur; Becton Dickinson, NJ, USA). For each individual, 100.000 leukocytes were acquired whilst live gating on leukocytes using forward and side-scatter characteristics. Data were saved for later analysis using FlowJo software (Tree star, Inc., Ashland, OR, USA).

**Figure 1:**
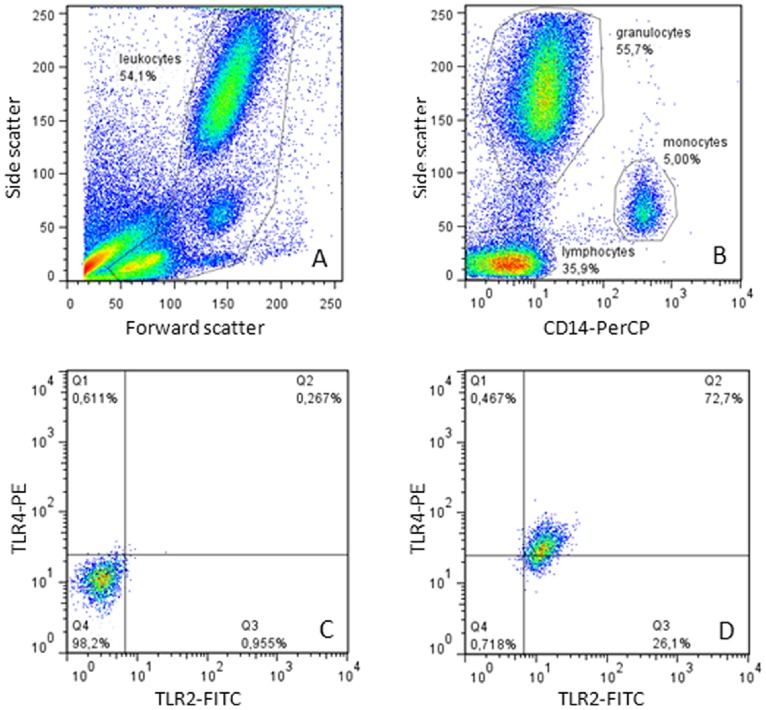
Gating strategy for leukocyte subpopulations and TLR expression. Leukocytes were selected in the forward-sidescatter plot (fig. 1A) and copied to a sidescatter-CD14 plot. Monocytes (CD14 positive cells), granulocytes (CD14 negative cells with high SSC) and lymphocytes (CD14 negative cells with low SSC) were gated (fig. 1B). CD14 positive cells were copied to a TLR2/TLR4 plot. Using the isotype control sample, gates were set in the TLR2/TLR4 plot so that at least 99% of the isotype controls were negative for TLR2/TLR4 expression (fig. 1C). This gate was then used to identify the percentages of TLR4/TLR2 double positive, TLR2 single and TLR4 single positive monocytes as well as their mean fluorescence intensity (MFI), in the antibody incubated samples (fig. 1D).

During analyses a gate was set on the leukocytes in the forward-sidescatter plot (fig. 1A). This gate was copied to a sidescatter-CD14 plot, in which monocytes (CD14 positive cells), granulocytes (CD14 negative cells with high SSC) and lymphocytes (CD14 negative cells with low SSC) were gated (fig. 1B). Total numbers of monocytes, granulocytes and lymphocytes were derived by multiplying the percentage of the subpopulations with the total WBC count (microcell counter model Sysmex pocH-100i Haematology Analyser, Sysmex Corp., Kobe, Japan). Thereafter, CD14 positive cells were copied to a TLR2/TLR4 plot. Using the isotype control sample, gates were set in the TLR2/TLR4 plot so that at least 99% of the isotype controls were negative for TLR2/TLR4 expression (fig. 1C). This gate was then used to identify the percentages of TLR4/TLR2 double positive, TLR2 single and TLR4 single positive monocytes as well as their mean fluorescence intensity (MFI), in the antibody incubated samples (fig. 1D).

### Data Analysis

All figures expressed individual results (line: mean or median, depending on normality of the data). Normality of the data was tested using the Kolmogorov-Smirnov test.

In the blood stimulation experiments, effects of the reproductive state (non-pregnant vs. pregnant) or effects of the bacteria or LPS species (*E-coli* vs Pg) were tested using two-way ANOVA followed by Bonferroni post-tests. In case data were not normally distributed, before using the two-way ANOVA, data were log transformed, which led to normal distribution of data.

For data on number of WBC and the differential cell counts and data on TLR expression, differences between pregnant and non-pregnant women in were evaluated using the Student’s T test. In all cases, the significance level was p<0.05.

## Results

### Basal Cytokine Concentrations in Whole Blood without Bacterial or LPS Stimulation

Basal cytokine concentrations in plasma (not stimulated with LPS or bacteria, but incubated at 37°C for 24 hr) are shown in table 1. It can be seen from this table that plasma TNFα is lower in pregnant women as compared with non-pregnant women. The concentrations of the other cytokines did not differ between pregnant and non-pregnant women.

**Table 1:** Basal levels of cytokines in plasma from pregnant and non-pregnant women.

	IL-1ß (ng/ml)(mean±sem)	IL-6 (ng/ml)(mean±sem)	IL-12 (ng/ml)(mean±sem)	TNFα (ng/ml)(mean±sem)	IL-10 (ng/ml)(mean±sem)
Pregnant	0.00674±0.00574	0.00325±0.00389	0.00060±0.00017	0.00145±0.00025*	0.00072±0.00012
Non-pregnant	0.00295±0.00101	0.00533±0.00480	0.00039±.00039	0.00382±0.00056	0.00052±0.00017

* significantly different from non-pregnant women (Students T-test, p<0.05).

### Whole Blood Cytokine Production following Bacterial Stimulation

**Figure 2:**
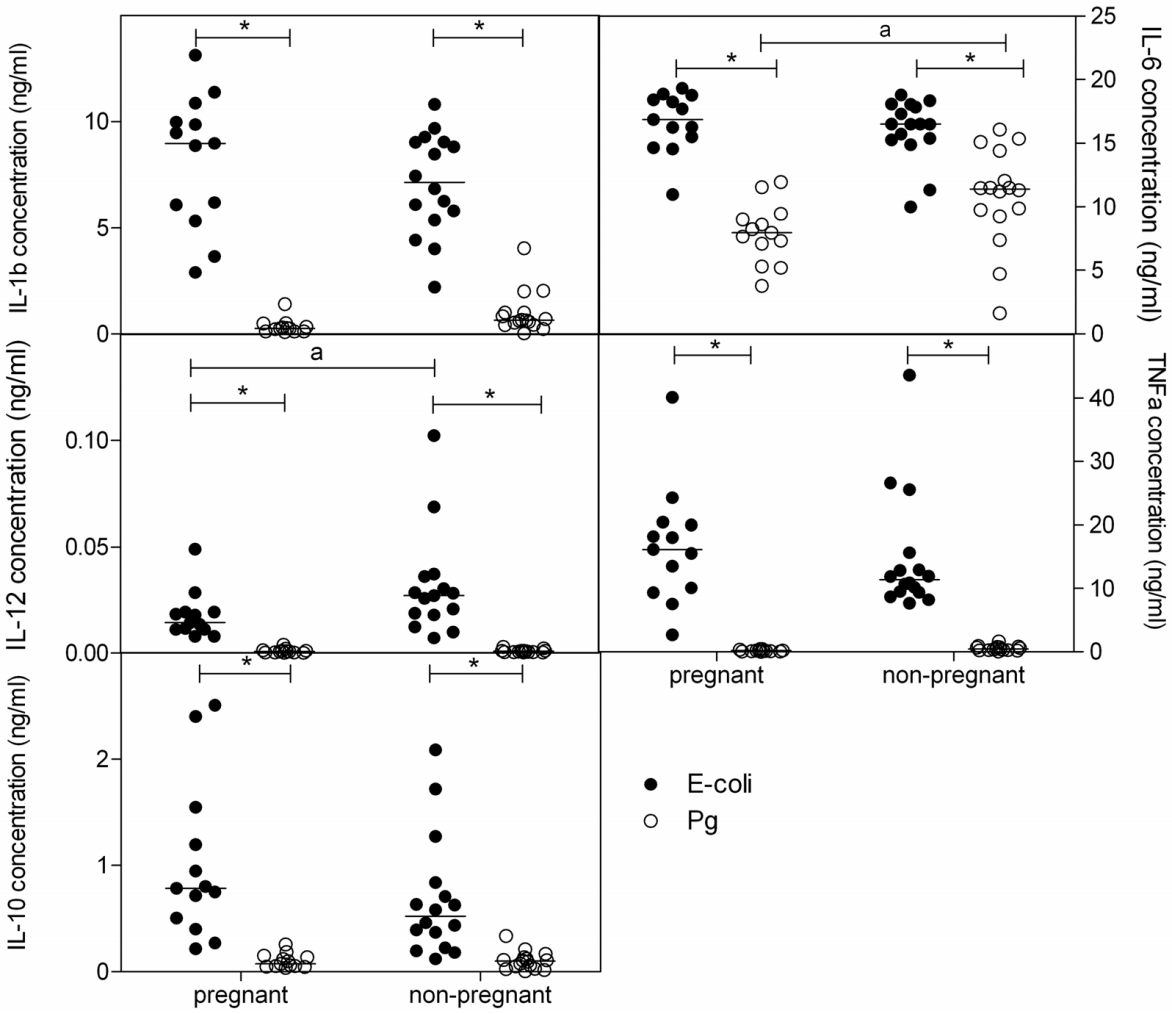
Cytokine concentrations following stimulation with bacteria. Concentrations (ng/ml) of IL-1b, IL-6, IL-12, TFNα and IL-10 in plasma of pregnant and non-pregnant women following stimulation of whole blood with *E-coli* (black dots) or *P. Gingivalis* (PG) (open dots) bacteria (5*10^7^ bacteria/ml) for 24 hr. *significantly different from *E-coli* (two-way ANOVA followed by Bonferroni post-tests, p<0.05)). a: significantly different from pregnant women after the same stimulation (two-way ANOVA followed by Bonferroni post-tests, p<0.05).

Figure 2 shows that for all cytokines tested, in pregnant and in non-pregnant women, *E-coli* bacteria induced a stronger cytokine production as compared with Pg bacteria (Two-way ANOVA and Bonferroni posttest, p<0.05). An effect of pregnancy was also observed: the concentration of IL-12 following *E-coli* stimulation was significantly lower in pregnant blood as compared with non-pregnant blood (p<0.05). The concentration of IL-6 following Pg stimulation was significantly lower (p<0.05) in pregnant blood vs. non-pregnant blood.

### Whole Blood Cytokine Production following LPS Stimulation

**Figure 3:**
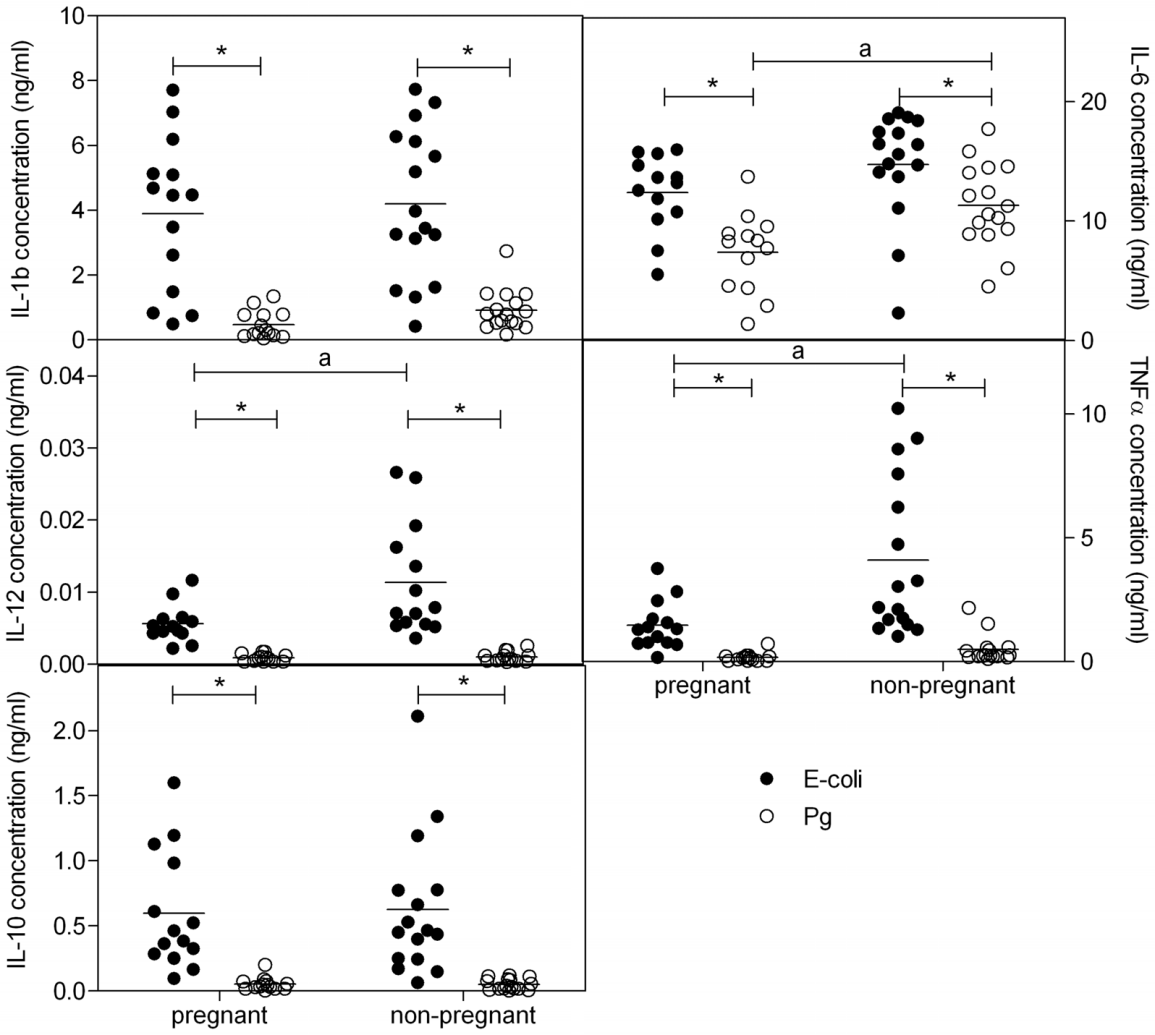
Cytokine concentrations following stimulation with LPS. Concentrations (ng/ml) of IL-1b, IL-6, IL-12, TFNα and IL-10 in plasma of pregnant and non-pregnant women following stimulation of whole blood with *E-coli* (black dots) or *P. Gingivalis* (PG) (open dots) LPS (2 µg/ml) for 24 hr. *significantly different from *E-coli* (two-way ANOVA followed by Bonferroni post-tests, p<0.05). a: significantly different from pregnant women after the same stimulation (two-way ANOVA followed by Bonferroni post-tests, p<0.05).

Also stimulation of blood of pregnant and non-pregnant women with *E-coli* LPS induced significantly higher production of all cytokines tested as compared with Pg LPS (Two-way ANOVA and Bonferroni posttest, p<0.05) (fig. 3). Moreover, concentrations of IL-12 and TNFα after stimulation with *E-coli* LPS were significantly lower in pregnant as compared with non-pregnant women. The concentration of IL-6 was significantly lower in pregnant vs non-pregnant women following stimulation with Pg LPS.

### Ratio of IL-12/IL-10, TNFα/IL-10, IL-6/IL-10

**Figure 4:**
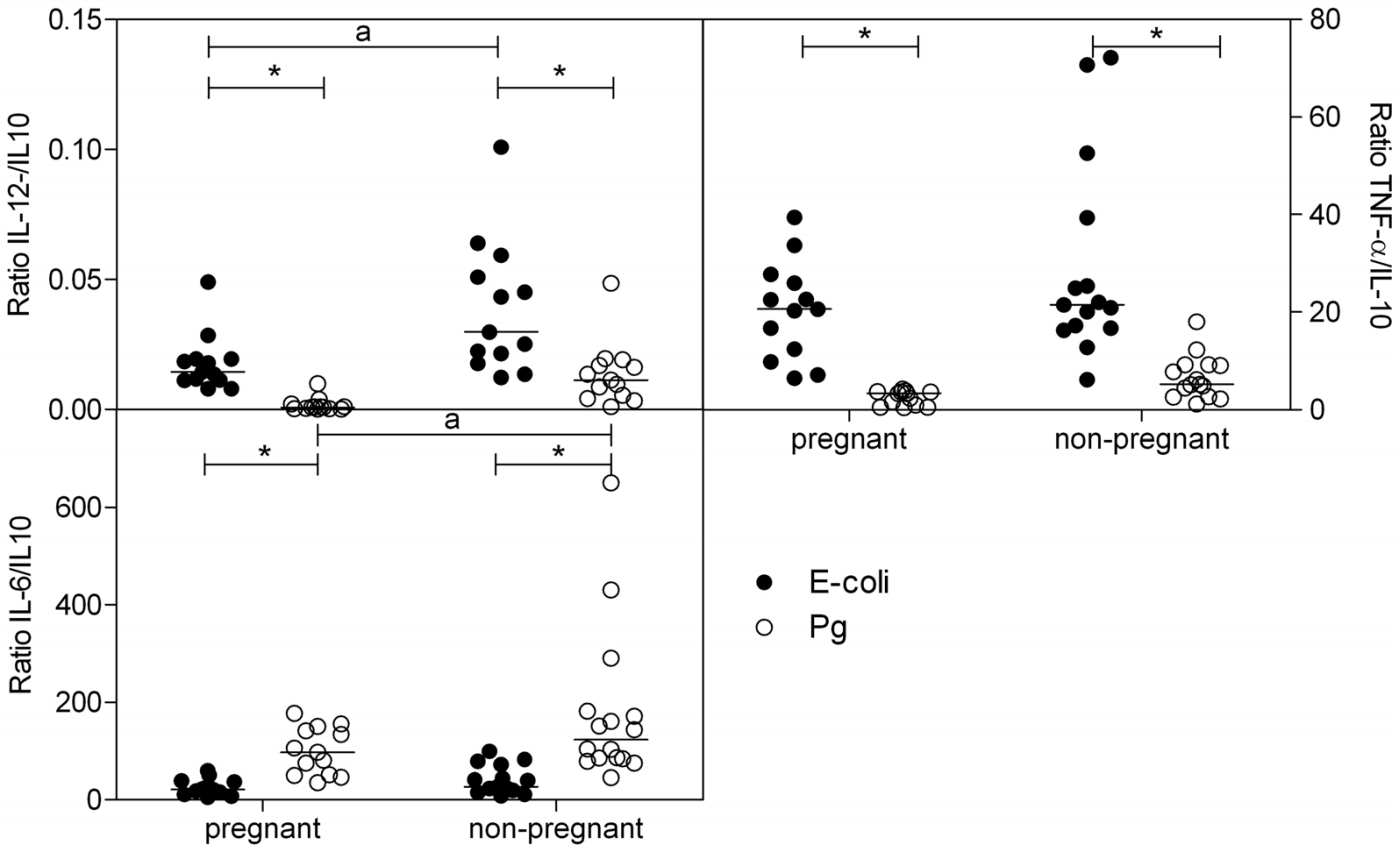
Pro-inflammatory/anti-inflammatory cytokine ratio following stimulation with bacteria. Ratio of Il-12/IL-10, TNFα/IL-10 and IL-6/IL-10 cytokine production in plasma of pregnant and non-pregnant women following stimulation of whole blood with *E-coli* (black dots) or *P. Gingivalis* (PG) (open dots) bacteria (5*10^7^ bacteria/ml) for 24 hr. *significantly different from *E-coli* (two-way ANOVA followed by Bonferroni post-tests, p<0.05). a: significantly different from pregnant women after the same stimulation (two-way ANOVA followed by Bonferroni post-tests, p<0.05).

Stimulation with *E-coli* bacteria resulted in a significantly higher IL-12/IL10, TNFα/IL-10 ratio and a significantly lower IL-6/IL10 ratio as compared with stimulation with Pg bacteria (fig. 4A) in both pregnant and non-pregnant women. Pregnant women showed a decreased IL-12/IL-10 ratio after stimulation with *E-coli* bacteria (fig. 4) and a decreased IL-6/IL-10 ratio following Pg bacterial stimulation as compared with non-pregnant women.

**Figure 5:**
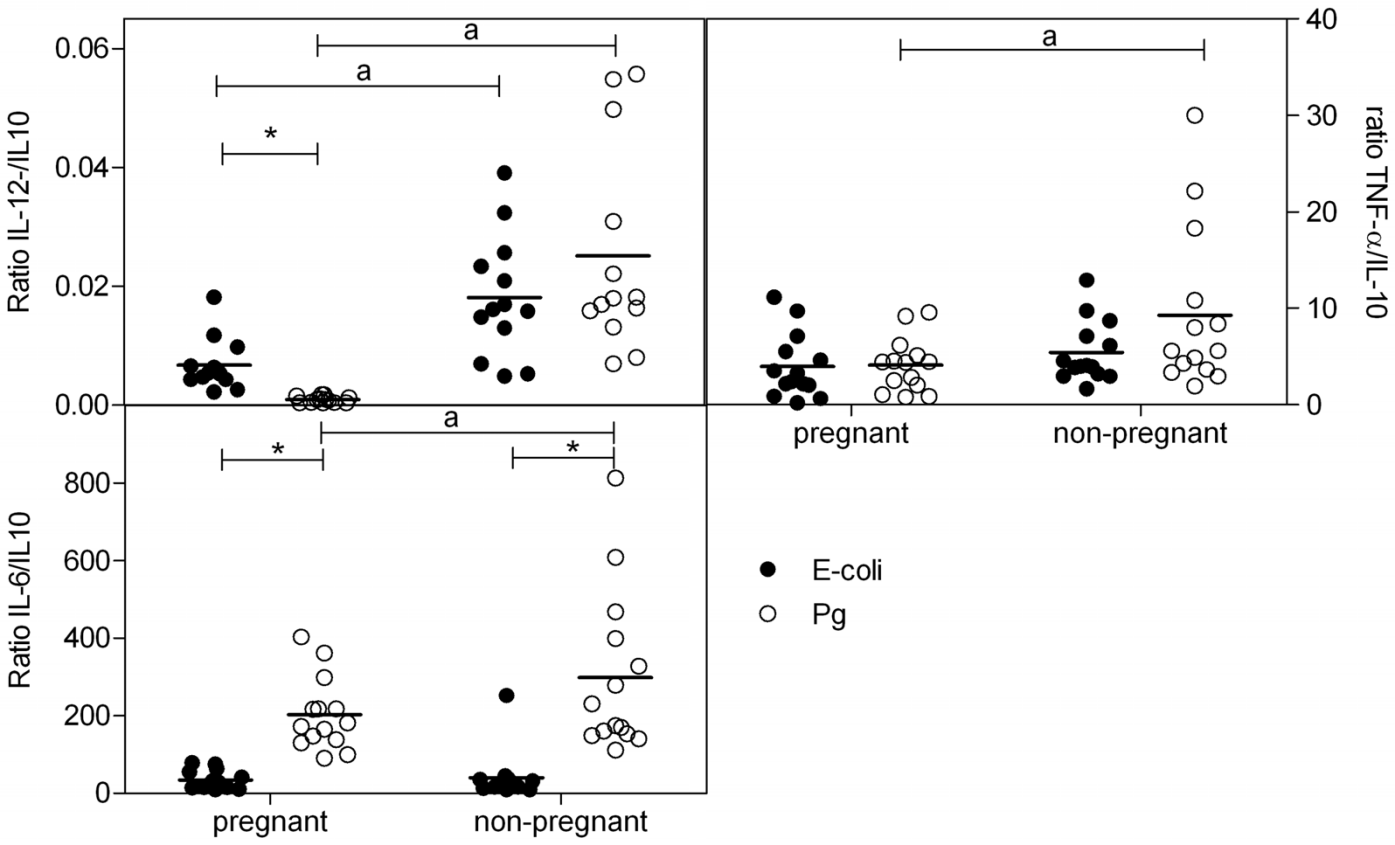
Pro-inflammatory/anti-inflammatory cytokine ratio following stimulation with LPS. Ratio of IL-12/IL-10, TNFα/IL-10 and IL-6/IL-10 cytokine production in plasma of pregnant and non-pregnant women following stimulation of whole blood with *E-coli* (black dots) or *P. Gingivalis* (PG) (open dots) LPS (2 µg/ml) for 24 hr. *significantly different from *E-coli* (two-way ANOVA followed by Bonferroni post-tests, p<0.05). a: significantly different from pregnant women after the same stimulation (two-way ANOVA followed by Bonferroni post-tests, p<0.05).

After LPS stimulation, we observed a higher IL-12/IL-10 ratio after *E-coli* LPS stimulation vs Pg LPS in blood of pregnant women and a lower IL-6/IL-10 ratio after *E-coli* LPS stimulation vs. Pg LPS stimulation in blood of both pregnant and non-pregnant women. The IL-12/IL10 ratio was decreased in pregnant vs. non-pregnant women for both types of LPS, while only for Pg LPS the TNFα/IL-10 and the IL-6/IL-10 ratio was decreased in pregnant vs. non-pregnant women (fig. 5).

### Changes in White Blood Cell Counts and TLR2 and TLR4 Expression in Pregnant Women

As cytokine production in the plasma may depend on the number of leukocytes, we measured WBC counts and percentages of leukocyte subsets in the blood samples (Table 2). A significant increase in total number of WBC, monocytes and granulocytes was seen during pregnancy as compared with the follicular phase (*p*<0.05, Student’s T test).

**Table 2:** Total white blood cell count and differential cell counts.

	WBC (*10^9^/L)(mean±SEM)	Granulocyte count(*10^9^/L) (mean±SEM)	Monocyte count (*10^9^/L) (mean±SEM)	Lymphocyte count (*10^9^/L)(mean±SEM)
Pregnant	9.96±0.62*	7.02±0.56*	0.62±0.07*	2.31±0.17
Non-pregnant	5.69±0.21	2.89±0.25	0.35±0.03	2.44±0.15

* significantly increased vs non-pregnant women (Student’s T test, p<0.05).

**Figure 6:**
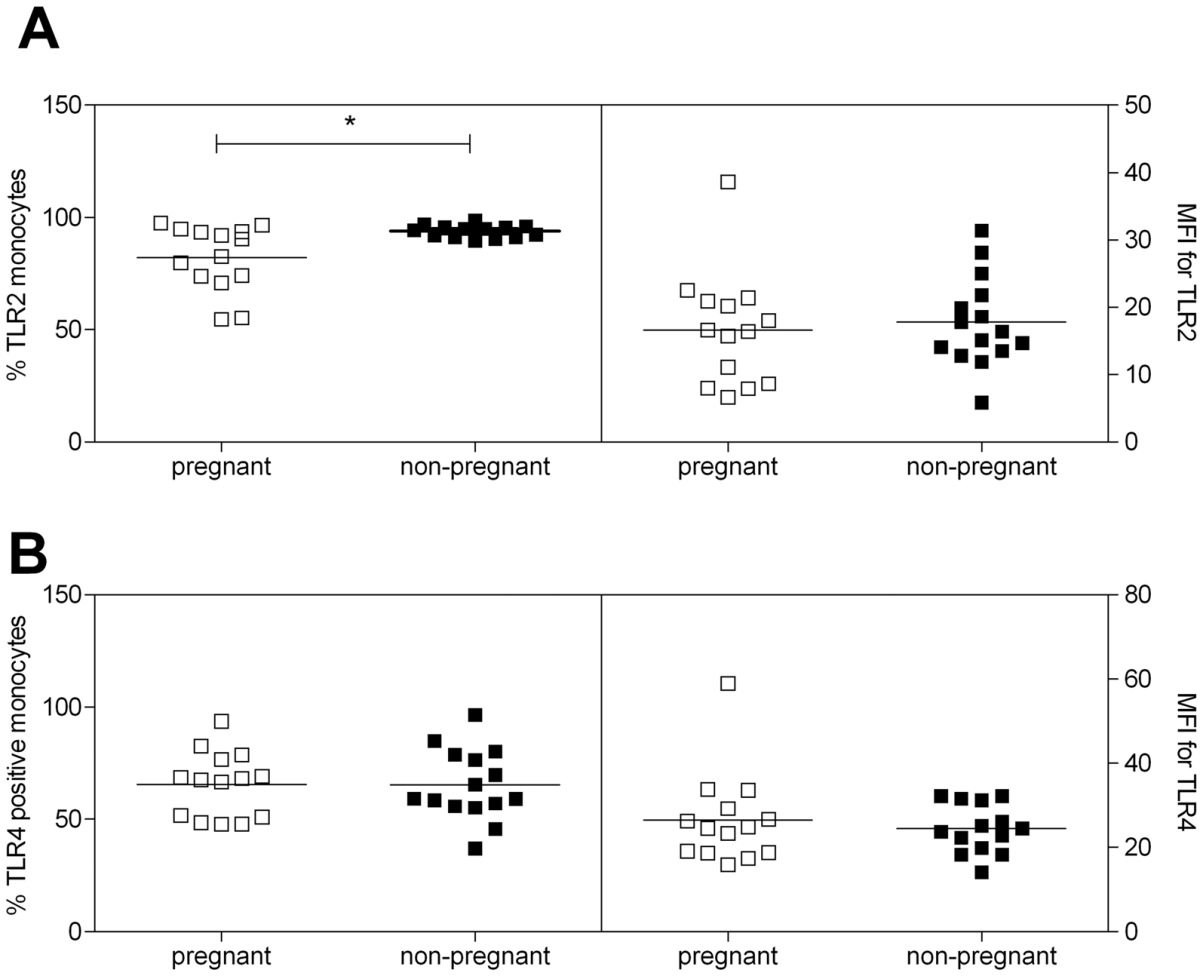
Expression of TLR2 and TLR4 in pregnant and non-pregnant women. Expression of TLR2 and TLR4 on monocytes of pregnant (open squares) and non-pregnant women (black squares). A: Percentage of TLR2 positive monocytes (left graph) and mean fluorescent intensity of TLR2 staining of monocytes (right graph). B: Percentage of TLR4 positive monocytes (left graph) and mean fluorescent intensity of TLR4 staining of monocytes (right graph). *: significantly increased vs pregnant women (student’s T test, p<0.05).

TLR are pattern recognition receptors, which are able to recognize bacteria and their products and induce an inflammatory response following recognition [22]. Since TLR2 and TLR4 are the main TLRs recognizing bacteria and LPS [22], we measured expression of these receptors on monocytes, the most important cells responsible for bacteria and LPS recognition. The percentage of TLR2^+^ monocytes decreased in pregnant vs. non-pregnant women (fig. 6A;Student’s T test, p<0.05), while the mean fluorescence intensity (MFI), a measure for expression of TLR2 per cell, was not affected by pregnancy.

The percentage TLR4^+^ monocytes and TLR4 MFI of monocytes was not different between pregnant and non-pregnant women (fig. 6B).

The percentage of double positive cells was also not affected by pregnancy (56.41±4.42 in pregnant vs. 57.85±4.48 in non-pregnant women; not shown).

## Discussion

The present study was conducted to evaluate the effect of pregnancy and different bacteria and their products on leukocyte cytokine production. We stimulated whole blood of pregnant and non-pregnant women with bacteria or LPS from *E-coli* or Pg. There was a generally lower cytokine production after stimulation with Pg bacteria or it’s LPS as compared with *E-coli* bacteria or it’s LPS in both non-pregnant and pregnant women. We also observed an effect of pregnancy upon cytokine production. In pregnant women the production of IL-6 upon Pg stimulation was decreased as compared with non-pregnant women, while the production of IL-12 and TNFα was decreased in pregnant women as compared with non-pregnant women following stimulation with *E-coli*. This illustrates that pregnancy affects cytokine responses upon Pg or *E-coli* stimulation differently and suggests that the varying responses during pregnancy upon different bacteria or their products may result from differences in cytokine production. The increased sensitivity of pregnant women to bacteria or their products may also result from differences in cytokine production.

We found a marked lower cytokine production and a relatively higher production of pro-inflammatory cytokines induced by Pg bacteria or LPS in comparison with *E-coli* bacteria or LPS in both pregnant and non-pregnant women. An important mechanism by which a decreased cytokine response upon LPS or bacterial stimulation could be explained is by decreased expression of pattern recognition receptors (PPR), amongst which Toll-like receptors (TLR) are the best studied [23]. These TLR recognize so-called PAMPs (pathogen-associated-molecules),; which arise from pathogens, and alarm an individual to intruding pathogens [24]. Similar changes in cytokine production were observed when comparing bacterial stimulation with LPS stimulation, this may suggest that LPS plays a large role in the cytokine production of whole blood after bacterial stimulation. Since LPS is recognized mainly by TLR2 (Pg LPS [25], [26]) and TLR4 (*E-coli* LPS [26]), we measured these 2 TLRs on the monocytes. Differences in expression between TLR2 and TLR4 on monocytes may result in different cytokine production following stimulation with these bacteria or LPS. However, despite the lower cytokine production after Pg bacteria or LPS, TLR2 is higher expressed by monocytes as compared with TLR4. Differences in TLR expression could also explain differences in responses of pregnant vs. non-pregnant women to Pg or *E-coli* LPS. We found a decreased expression of TLR2 on monocytes of pregnant vs. non-pregnant women, with no changes in TLR4 expression. Although production of some cytokines were decreased during pregnancy after stimulation with Pg LPS, this was not the case for all cytokines. The role of other bacterial products which are recognized by other TLR, such as flagelin (TLR 5) or bacterial DNA (TLR 9), in the production of cytokines during pregnancy is subject of further investigation.

The finding that cytokine production after stimulation with Pg bacteria or LPS is generally lower as compared with stimulation with *E-coli* bacteria or LPS in non-pregnant women is in line with previous studies [21], [27]. Our study for the first time shows these differences in pregnant women. Such lower cytokine production and lower pro-inflammatory cytokine ratio following stimulation with Pg LPS, as compared with *E-coli* LPS, may be involved in the in vivo differences in responses of pregnant animals to these LPS species: while *E-coli* LPS induces a preeclampsia-like syndrome in pregnant rats [18], Pg LPS only induced hypertension in pregnant rats [19]. Apparently, a preeclampsia-like syndrome is induced by pro-inflammatory cytokines, such as for instance TNFα. This cytokine, indeed also induced a preeclampsia-like syndrome in pregnant rats [28].

In the present study we have chosen doses of bacteria and LPS that induced maximal cytokine production. We don’t expect that other concentrations would have shown different results. This suggestion is based on 2 observations: A previous study from our lab [21] showed that stimulation of a monocyte cellline with various doses of *E-coli* or Pg bacteria resulted in higher TNFα production after *E-coli* stimulation vs Pg stimulation at all concentrations tested. Similar results were found for LPS stimulation. Therefore the differences between *E-coli* and Pg bacteria or LPS stimulation seem not to depend on the doses used. Also the effect of pregnancy, appears not to be dependent on the dose used. This statement is based on unpublished pilot studies from our lab, in which we tested various concentrations of *E-coli* LPS (ranging from 0 until 2 µg/ml) on monocyte TNFα production from pregnant and non-pregnant women (using flow cytometry). In both groups of women, very little TNFα was produced at concentration of 2×10^−5^ µg/ml LPS, while maximum responses were observed after 5×10^−2^ µg/ml. Decreased production of TNFα in pregnant vs non-pregnant women were already observed at concentrations of 2×10^−4^ µg/ml of LPS, and the maximal difference was observed after maximal stimulation.

The differences in IL-6 production between the two strains, are smaller as compared with the production of other cytokines. This is reflected in an increased IL-6/IL-10 ratio following Pg bacteria or LPS stimulation as compared with *E-coli* bacteria or LPS stimulation. It may be important for Pg bacteria to induce relatively high levels of IL-6, since IL-6 plays an important role in periodontal disease. IL-6 is an important cytokine with diverse functions. It regulates the immune response and leukocyte recruitment [29], but can also affect bone formation [30]. It has also been shown that IL-6 has potent anti-inflammatory properties, as it can inhibit the production of TNFα [31] and can increase the production of IL-10 and IL-1ra [32]. Therefore the relatively high production of IL-6 induced by stimulation with Pg bacteria or LPS may, next to the relatively low overall cytokine production, be involved in the different response of women to these bacteria or its LPS.

Interestingly, despite the fact that pregnant individuals are much more sensitive to LPS, the production of cytokines following LPS (of both species) stimulation is either similar or decreased in pregnant women as compared with non-pregnant women. This suggests that pregnant women may be more sensitive to the effects of these cytokines. This is in line with earlier results from our lab [33]. If results would have been presented as amount of cytokines per monocyte, the differences would even be more extreme (results not shown), since the number of monocytes is increased in blood of pregnant women, indicating that monocytes of pregnant women produce less cytokines upon a similar LPS or bacterial stimulus than monocytes of non-pregnant women [13]. Such a decreased production of cytokines by pregnant monocytes may be due to their increased activational status: monocytes of pregnant women show increased CD14, CD11b and CD64 expression and decreased CD62L expression [12]. This may result in an endotoxin tolerant state, similar to the “endotoxin tolerance” seen in monocytes from septic patients [34], in which monocytes are less able to produce cytokines. Interestingly, basal production of TNFα, but not of the other cytokines, was lower in pregnant women as compared with non-pregnant women. Since also these samples have been incubated for 24hr, some monocyte activation may have occurred during the incubation and the decreased TNFα production in pregnant women may have been due to a similar mechanism of endotoxin tolerance.

In summary, the generally lower production of cytokines as well as the decreased proinflammatory ratio after Pg stimulation vs *E-coli* stimulation in pregnant women may be responsible for the differences in the in vivo response upon the bacteria and their products in these women. Although pregnant women are extremely sensitive to LPS, the production of IL-12, TNFα and IL-6 upon stimulation with bacteria or LPS were decreased, suggesting that pregnant women are more sensitive to these cytokines. The mechanism of decreased cytokine production remains unknown from this study, but it may be related to decreased NF-kB expression, which is an important transcription factor for proinflammatory cytokine production [35], and which is decreased pregnancy [36], [37]. The exact mechanism of decreased cytokine production during pregnancy requires further investigation.
